# A novel autophagy inhibitor, bTBT, disturbs autophagosome formation

**DOI:** 10.1080/27694127.2023.2194620

**Published:** 2023-04-06

**Authors:** Momoka Chiba, Mai Yanagawa, Yurika Oyama, Shingo Harada, Tetsuhiro Nemoto, Akira Matsuura, Eisuke Itakura

**Affiliations:** a Department of Biology, Graduate school of Science and Engineering, Chiba University; b Graduate School of Pharmaceutical Sciences, Chiba University; c Department of Biology, Graduate School of Science, Chiba University, Chiba, Japan

**Keywords:** autophagy, inhibitor, WIPI2, autophagosome formation, bis-tributyltin

## Abstract

Macroautophagy (hereafter, autophagy) is a form of intracellular degradation in which autophagosome formation is systematically coordinated by multiple processes involving numerous autophagy-related gene (ATG) proteins. Autophagy-modulating compounds are valuable for understanding the molecular mechanism of autophagy and its clinical application. Although several autophagy inhibitors have been identified, their inhibitory steps during autophagosome formation by the inhibitors are limited. Herein, we identified a novel autophagy inhibitor, bis-tributyltin (bTBT), which inhibits a unique step in autophagosome formation. In mammalian cells, bTBT treatment suppresses LC3 flux and accumulates most of ATG proteins, including LC3 and early ATG proteins (ULK1, ATG16L1, and WIPI2), in punctate structures. On the other hand, LAMP1, a lysosomal marker, did not co-localize with accumulated LC3 after bTBT treatment, indicating bTBT inhibits a late step of autophagosome formation. Stx17, a soluble *N*-ethylmaleimide-sensitive factor attachment protein receptor protein that mediates autophagosome–lysosome fusion, is usually recruited to LC3-positive structures after the dissociation of early ATG proteins. However, bTBT accumulates Stx17 and WIPI2 positive large autophagic structures and maintains the autophagic structures for much longer. In conclusion, we identified a novel type of autophagy inhibitor, bTBT, which disturbs autophagosome formation.

## Abbreviations

ATG, autophagy-related gene; bTBT, bis-tributyltin; LC3, microtubule-associated protein 1A/1B-light chain 3; MEFs, mouse embryonic fibroblasts; PI3P, phosphatidylinositol 3-phosphate; Stx17, syntaxin 17; ULK1, Unc-51-like autophagy activating kinase1; VPS34, vacuolar sorting protein 34

## Introduction

Autophagy is an intracellular degradation process. A membrane structure called the phagophore /isolation membrane elongates and sequesters intracellular substrates such as proteins and organelles. The membrane closes to form a double membrane structure called the autophagosome. Autophagosomes then fuse with lysosomes, and the enclosed substrates are degraded by lysosomal degradation [1,2].

Autophagy is responsible for maintaining cellular homeostasis, and it plays an essential role in removing unwanted or toxic substances via degradation or recycling of nutritional resources within the cell. Thus, autophagy is involved in various diseases [3]; for example, impaired autophagy is associated with neurodegenerative and infectious diseases [4], while activated autophagy provides nutrients for cancer cells during the proliferation phase, allowing excessive growth [5]. Therefore, autophagy is a promising target for the treatment of various diseases [6].

Autophagosome formation is systematically regulated by multiple processes involving dozens of autophagy-related gene (ATG) proteins [7]. After autophagy induction in mammals, the phagophore is generated and elongated by a hierarchical mechanism involving early ATG proteins such as Unc-51-like autophagy activating kinase1 (ULK1), ATG9A, ATG14, vacuolar protein sorting 34 (VPS34), WIPI, ATG12, and ATG16L1 [8]. First, the ULK1 complex and ATG9A vesicles assemble at the autophagosome formation site [9]. Subsequently, ATG14 and the phosphatidyl inositol 3-kinase (PI3K) VPS34 complex produces phosphatidylinositol 3-phosphate (PI3P) on the membrane [10]. PI3P recruits the ATG2–WIPI complex, which supplies lipids to the phagophore [11]. Then, the ATG12–ATG5–ATG16L1 complex, recruited to the phagophore by ATG2–WIPI, induces lipidation of microtubule-associated protein light chain 3 (LC3) to generate LC3- phosphatidylethanolamine (PE) on the phagophore [12]. LC3 alters bilayer curvature and induces hemifusion for autophagosome closure [13]. Early ATG proteins, excluding LC3, dissociate from the autophagosome before the closure step [14,15]. Finally, syntaxin 17 (Stx17) is recruited to the autophagosome just before or after autophagosome closure [14,16]. The completed autophagosome fuses with lysosomes via Stx17, forming autolysosomes and leading to degradation of the enclosed materials.

Several autophagy-modulating compounds have been identified, providing a better understanding of the molecular mechanisms of autophagy, and some have been used in clinical trials [17]. For example, wortmannin, a PI3K activity inhibitor, suppresses autophagy at an early step by inhibiting VPS34 [18]. Bafilomycin A_1_ and chloroquine inhibit autophagic activity by preventing autophagosome–lysosome fusion and acidification inside lysosomes [19]. Hydroxychloroquine, a hydroxyl analog of chloroquine, is effective as a cancer therapy when co-administered with anticancer drugs [20]. As described above, numerous ATG proteins are involved in multiple steps of autophagosome formation. However, known inhibitory steps in autophagosome formation by inhibitors are limited [21]. Therefore, identifying a novel inhibitor that modulates the different steps of autophagy could provide new insights into the molecular mechanisms of autophagy and broaden the potential clinical applications of autophagy.

In this study, we report a novel autophagy inhibitor, bTBT, that blocks a unique step in autophagosome formation. Unlike known autophagy inhibitors, the inhibitor bTBT accumulates large autophagic structures with most of autophagy proteins, including ULK1, WIPI2, LC3, and Stx17, and reduces autophagic activity in mammalian cells. Based on the results, we have proposed that bTBT is a novel autophagy inhibitor that retards the formation of completed autophagosomes.

## Results

### bTBT reduces autophagic activity

**Figure 1.**
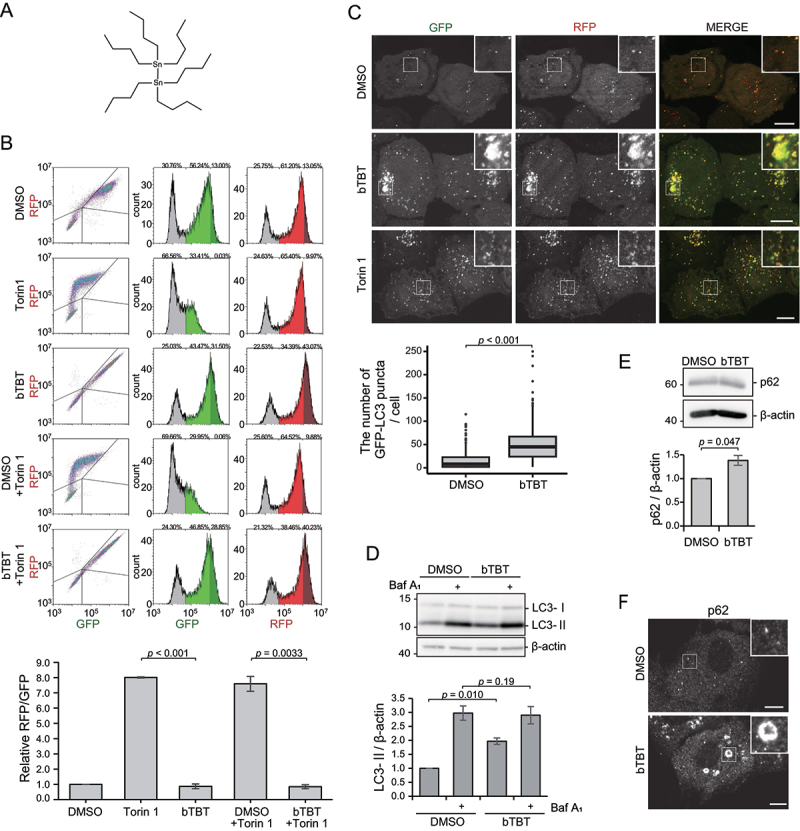
**bTBT is a novel autophagy inhibitor** (**A**) Structural formula of bis-tributyltin (bTBT). (**B**) bTBT inhibits lysosomal degradation of RFP-GFP-LC3. HeLa cells expressing RFP-GFP-LC3 were cultured with DMSO, bTBT, or Torin 1 for 18 h and analyzed by flow cytometry. The bar plot represents the mean ± SE of three independent experiments. *p*-values were calculated by Welch’s *t*-test.(**C**) bTBT increases the number of punctate structures positive for RFP-GFP-LC3. HeLa cells expressing RFP-GFP-LC3 were cultured with DMSO, bTBT, or Torin 1 for 2 h and then fixed. Fluorescence images were obtained using a confocal microscope. Scale bars, 10 µm. The box-and-whisker plot shows the number of GFP-LC3 punctures, which were counted from more than 150 cells. *p*-value was calculated by Welch’s *t*-test. (**D**) bTBT induces LC3-II accumulation. HeLa cells were cultured with DMSO or bTBT for 4 h. Cells were lysed in lysis buffer and analyzed by immunoblotting using anti-LC3 and anti-β-actin antibodies. The bar plot represents the mean ± SE of three independent experiments. *p*-values were calculated by Welch’s *t*-test.(**E**) bTBT induces p62 accumulation. An immunoblot derived from MEFs cultured with DMSO or bTBT for 18 h is shown. Cells were lysed in lysis buffer and analyzed by immunoblotting using anti-p62 and anti-β-actin antibodies. Scale bars, 10 µm. The bar plot represents the mean ± SE of three independent experiments. *p*-value was calculated by Welch’s *t*-test.(**F**) bTBT induces accumulation of large p62-positive punctate. MEFs were cultured with DMSO or bTBT for 2 h. Then, cells were fixed and stained with anti-p62 antibody. Immunofluorescence images were obtained using a confocal microscope. Scale bars, 10 µm.

We performed compound screening using an autophagic flux assay, and bTBT was identified as a potential autophagy inhibitor (Figure 1A). To investigate the effect of bTBT on autophagic activity in mammalian cells, autophagic activity was quantified using flow cytometry and tet-on HeLa cells expressing the autophagic flux probe RFP-GFP-LC3 [22]. Once RFP-GFP-LC3 reaches the lysosome, GFP fluorescence is diminished by the lysosomal proteases and low pH inside the lysosome; however, RFP is resistant to them and accumulates in the lysosome. Therefore, the GFP/RFP fluorescence ratio serves as an indicator of autophagy degradation activity. Treatment with Torin 1, an autophagy inducer, reduced GFP but not RFP fluorescence (Figure 1B). Conversely, in bTBT-treated cells, GFP intensity was increased and the RFP/GFP fluorescence ratio decreased compared with DMSO-treated cells (Figure 1B). Furthermore, co-treatment with bTBT and Torin 1 suppressed the reduction in GFP fluorescence intensity (Figure 1B), indicating that bTBT inhibits autophagic flux under basal conditions and mTOR-inhibition dependent autophagy.

Next, we examined the subcellular localization of RFP-GFP-LC3, represented by GFP^+^RFP^+^ (yellow) puncta in autophagosomes and GFP^−^RFP^+^ (red) puncta in autolysosomes. Compared with DMSO, bTBT markedly increased the number of yellow puncta, whereas Torin 1 increased that of both yellow and red puncta. In addition, bTBT-induced yellow puncta were larger than the puncta observed in DMSO- or Torin 1-treated cells (Figure 1C). This result indicates that bTBT accumulates autophagosomal structures even under basal conditions.

An inhibitory effect of bTBT was also confirmed by biochemical analyses. Immunoblot analysis revealed that bTBT treatment increased the LC3-II level (autophagosome-associated lipidated form) compared with DMSO treatment (Figure 1D). Co-treatment with bafilomycin A_1_ and bTBT failed to synergistically increase the LC3-II level compared with bafilomycin A_1_ and DMSO co-treatment, indicating impaired autophagic flux. SQSTM1/p62 (sequestosome 1) is a substrate for autophagy. We found that the level of p62 was increased by bTBT (Figure 1E). Immunofluorescence showed an increase of p62 puncta and accumulation of larger p62-positive puncta in bTBT-treated cells compared with DMSO-treated cells (Figure 1F). These data indicate that aberrant autophagic substrates accumulated after bTBT treatment, and that bTBT inhibits autophagic degradation.

### bTBT inhibits the late step of autophagosome formation

**Figure 2.**
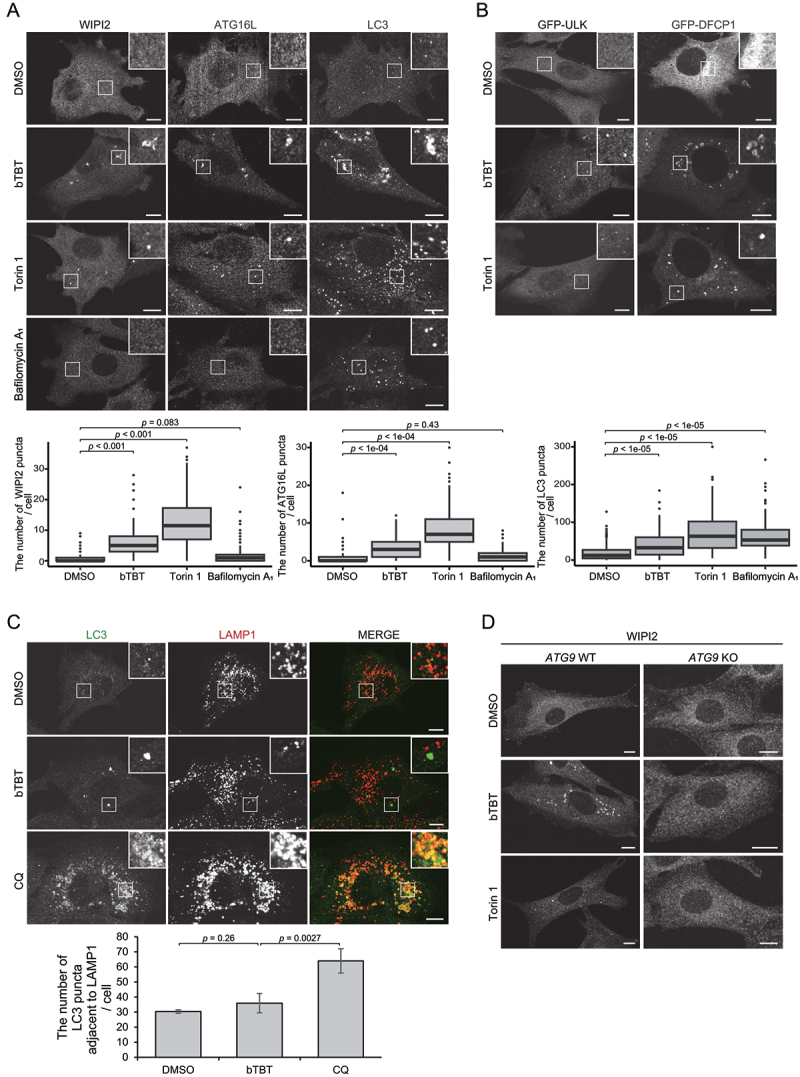
**bTBT induces accumulation of autophagosomal structures along with early ATG proteins** (**A**) bTBT induces accumulation of early ATG-positive punctate structures. MEFs were cultured with DMSO, bTBT, Torin 1, or bafilomycin A_1_ for 2 h. Then cells were fixed and stained with the indicated antibodies. Immunofluorescence images were obtained using a confocal microscope. Scale bars, 10 µm. The box-and-whisker plots show the number of punctures for each ATG protein, which were counted from more than 150 cells. *p*-values were calculated by Dunnett’s test. (**B**) bTBT induces the accumulation of early ATG-positive punctate structures. MEFs expressing GFP-ULK1 or GFP-DFCP1 were cultured with DMSO, bTBT, Torin 1, or bafilomycin A_1_ for 2 h. Then cells were fixed and stained with the indicated antibodies. Immunofluorescence images were obtained using a confocal microscope. Scale bars, 10 µm. (**C**) LC3 puncta induced by bTBT does not co-localize on the lysosomes. MEFs were cultured with DMSO, bTBT, or Chloroquine (CQ) for 2 h and then fixed and stained with the indicated antibodies. Immunofluorescence images were obtained using a confocal microscope. Scale bars, 10 µm. The numbers of co-localizations were analyzed by image J. The bar represents the mean ± SE of the number of LC3 puncta adjacent to LAMP1 of three independent experiments (n > 30 cells in each experiment). *p*-values were calculated by Welch’s *t*-test. (**D**) bTBT-induced autophagic structures are not observed in *ATG9* KO MEFs. *ATG9* WT or *ATG9* KO MEFs were cultured with DMSO, bTBT, or Torin 1 for 2 h. Then, cells were fixed and stained with anti-WIPI2 antibodies. Immunofluorescence images were obtained using a confocal microscope. Scale bars, 10 µm.

The hierarchical assembly of ATG proteins (ULK1 complex, WIPI and DFCP1 [23], ATG16 complex, LC3, and Stx17) drives autophagosome formation [8,16]. To determine which step of autophagosome formation that bTBT inhibits, ATG proteins recruited to autophagosomal structures, including DFCP1, ULK1, WIPI2, ATG16L1, and LC3, were investigated using immunofluorescence using mouse embryonic fibroblasts (MEFs) expressing GFP-fused ATG proteins. All of the observed proteins accumulated in large punctate structures in bTBT-treated cells (Figure 2A and 2B). Notably, many of the larger punctate structures were ring- or cup-like structures, which was hardly observed in Torin 1-treated cells. In addition, the accumulated LC3 induced by bTBT treatment was not colocalized with LAMP1, whereas with chloroquine treatment (chloroquine is a lysosomal inhibitor), co-localization of LC3 with LAMP1 significantly increased (Figure 2C). These results indicate that bTBT mainly inhibits a late step in autophagosome formation before lysosomal fusion step.

The accumulated structures in bTBT-treated cells were possibly aggregates that formed independently of autophagy. To confirm whether these puncta are autophagosomal structures, WIPI2 in *FIP200*- and *ATG9*-knockout (KO) MEFs were evaluated. FIP200 and ATG9 are involved in an early step of autophagosome formation. *ATG9*-KO and *FIP200*-KO cells fail to recruit most ATG proteins [8,9]. After bTBT treatment, WIPI2 was not observed in any punctate structures in *ATG9*-KO and *FIP200*-KO cells (Figure 2D and Supplemental Figure 1), indicating that WIPI2-positive structures accumulating after bTBT treatment are autophagosomal structures.

### bTBT inhibits autophagy differently compared with tubulin inhibitors

**Figure 3.**
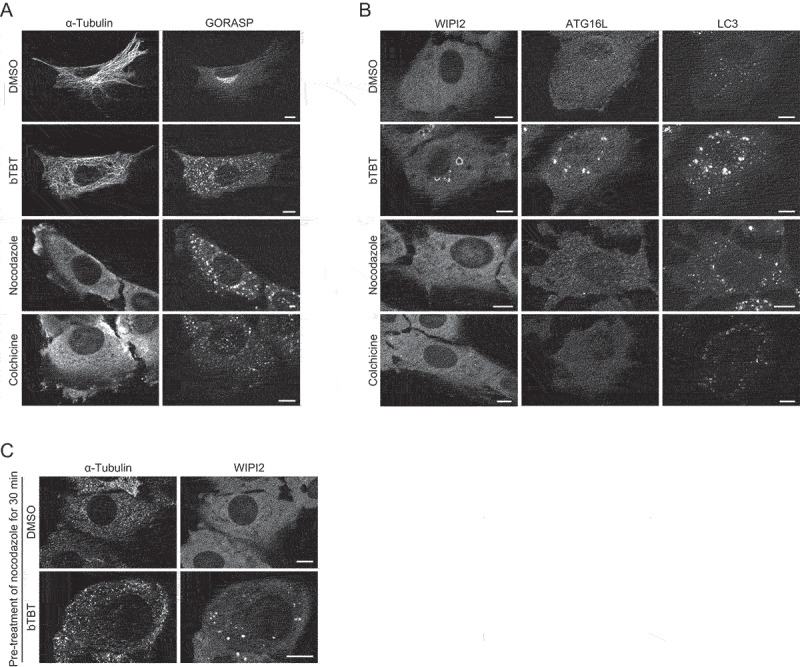
**bTBT independently inhibits autophagy and microtubule structure** (**A** and **B**) bTBT induces abnormal microtubule structures and Golgi fragmentation. MEFs were cultured with DMSO, bTBT, nocodazole, or colchicine for 2 h. Then, cells were fixed and stained with anti-α-tubulin, anti-GORASP2, anti-WIPI2, anti-ATG16L1, or anti-LC3 antibodies. Immunofluorescence images were obtained using a confocal microscope. Scale bars, 10 µm. **(C)** Abnormal tubulin structures are not associated with the accumulation of WIPI2 puncta in bTBT-treated cells. MEFs were cultured with nocodazole for 30 min followed by incubation with DMSO or bTBT for 2 h. Then, cells were fixed and stained with anti-α-tubulin or anti-WIPI2 antibodies. Immunofluorescence images were obtained using a confocal microscope. Scale bars, 10 µm.

After overnight treatment with bTBT, the cells developed a round shape and had detached from the culture dish (data not shown). Therefore, we treated the cells with bTBT for 2 h in most of experiments. Because autophagy deficiency is generally not cytotoxic to cells, bTBT might have another effect on cells; thus, several organelles and cytoskeletal components were evaluated (data not shown). Treatment with bTBT induced an aberrant curved tubulin structure (Figure 3A). In addition, the Golgi apparatus formed along microtubules was fragmented in bTBT-treated cells (Figure 3A). Because microtubules are essential for transporting lysosomes and autophagosomes, tubulin polymerization inhibitors, such as nocodazole or colchicine, decrease autophagic flux [24,25]. Thus, bTBT possibly also inhibits autophagy by hindering microtubule formation. Tubulin polymerization inhibitors led to accumulation of LC3 only; however, bTBT led to accumulation of other ATG proteins including WIPI2 and Atg16L1 (Figure 3B). Furthermore, accumulation of large WIPI2 puncta was observed in cells pre-treated with nocodazole prior to bTBT treatment (Figure 3C). Therefore, these data indicate that bTBT inhibits both autophagosome formation and microtubule formation independently.

### bTBT induces accumulation of aberrant autophagic structures

**Figure 4.**
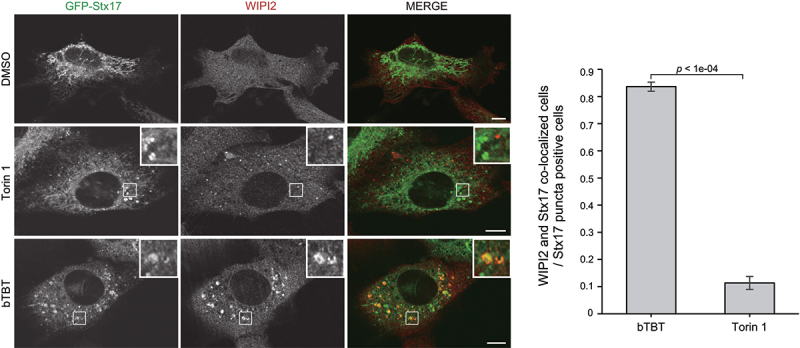
**bTBT induces accumulation of early and late autophagic markers simultaneously** MEFs stably expressing GFP-Stx17 were cultured with DMSO, bTBT, or Torin 1 for 2 h. The cells were then fixed and stained with an anti-WIPI2 antibody. Immunofluorescence images were obtained using a confocal microscope. Scale bars, 10 µm. The bar plot represents the mean ± SE of the ratio of the puncta co-localized with WIPI2 and Stx17 to Stx17 puncta-positive cells. Three independent experiments were performed (n > 40 cells in each experiment). *p*-value was calculated by Welch’s *t*-test.

Stx17 is recruited to the autophagosome and mediates autophagosome-lysosome fusion. Under normal conditions, Torin 1-induced GFP-Stx17-positive puncta do not co-localize with early ATG proteins including WIPI2 (Figure 4A) [16]. However, bTBT treatment induced GFP-Stx17-positive puncta that were larger and were co-localized with WIPI2 (Figure 4A). Early autophagy proteins in normal cells are localized only on the phagophore and dissociate before autophagosome completion, and Stx17 is recruited immediately before or after autophagosome completion [14], indicating that early ATG proteins coexisting with Stx17 in bTBT-treated cells represent aberrant autophagosomal structure. Alternatively, Stx17 is involved in the earliest steps of autophagosome biogenesis [26-28]. Thus, bTBT may disturb membrane biogenesis at the autophagosome formation site.

**Figure 5.**
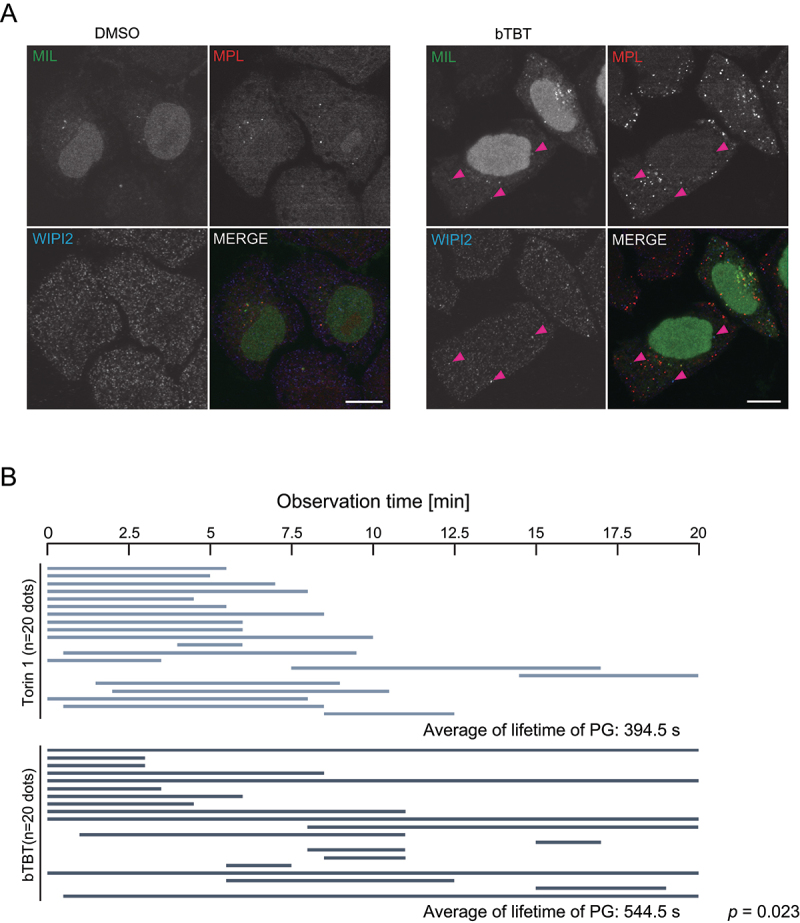
**bTBT-induced autophagic structures contain WIPI2-positive closed autophagosomes** (**A**) HeLa cells stably expressing Halotag-LC3 were cultured with DMSO or bTBT for 2 h and subjected to the Halotag-LC3 autophagosome completion assay followed by confocal microscopy. LC3 signals in the phagophore or outer autophagosomal membrane and in the autophagosome lumen were stained with Alexa Fluor 488-conjugated MIL and tetramethyl rhodamine (TMR)-conjugated MPL, respectively. White arrowheads indicate WIPI2-positive autophagic structures. (**B**) The lifetime of GFP-WIPI2-positive autophagic structures is longer in bTBT-treated cells than in Torin 1-treated cells. MEFs stably expressing GFP-WIPI2 were cultured with Torin 1 or bTBT for 4 h and then observed for 40 min under a confocal microscope. *p*-value was calculated by pearson’s chi-squared test for life time of GFP-WIPI2 between Torin 1- and bTBT-treated cells (n > 20).

To analyze whether the autophagosomal membrane that accumulates after bTBT treatment remains unclosed, an autophagosomal completion assay was conducted using HeLa cells expressing HaloTag-LC3 [29]. The assay used two HaloTag ligands: membrane-impermeable Alexa Fluor 488 HaloTag ligand (MIL) and membrane-permeable tetramethyl rhodamine HaloTag ligand (MPL). Cells were treated with MIL followed by MPL. MIL^+^MPL^−^ structures represented unclosed phagophores, MIL^+^MPL^+^ structures represented nascent autophagosomes, and MIL^−^MPL^+^ structures represented mature autophagosomes and autolysosomes. All of these structures were observed in cells treated with bTBT (Figure 5A). This result is consistent with the result that bTBT treatment accumulated LC3 puncta with or without ATG16L (Figure 2 and Supplemental Figure 2), suggesting that bTBT leads to the accumulation of both unclosed and closed structures. Notably, WIPI2 co-localized only with MIL^+^MPL^−^ structures, indicating that WIPI2-positive structures accumulated by bTBT treatment are unclosed phagophores. This is consistent with the observation that cup-like structures were induced by bTBT treatment (Figure 2); thus, bTBT might retard the formation of the completed autophagosome.

To investigate whether bTBT retards autophagosome formation, we examined the WIPI2 kinetics in bTBT-treated cell using live imaging of GFP-WIPI2. GFP-WIPI2-positive structures were maintained for an average of 394.5 s in the Torin 1-treated cell (Figure 5B and Movie S1). In contrast, the lifetime of some of the large GFP-WIPI2-positive structures in bTBT-treated cell was found to be much longer (average 544.4 s) than that in Torin 1-treated cells. This result suggests that bTBT may delay the dissociation of WIPI2 from the phagophores, which disturbs the completion of autophagosomes.

## Discussion

In this study, we identified bTBT as a novel autophagy inhibitor that reduces autophagic flux in a unique manner. bTBT treatment induced accumulation of excessive autophagic structures containing early ATG proteins, which are suppressed at a late step of autophagosome formation. Furthermore, bTBT treatment caused abnormal microtubule structures (Figure 3A) and potentially generated partial depolymerization of microtubules, inhibiting autophagosome–lysosome fusion. Notably, disruption of microtubules caused by nocodazole before bTBT treatment did not prevent the accumulation of early ATG proteins induced by bTBT (Figure 3C), indicating that disturbance of ATG proteins by bTBT is independent of microtubule structure. Taken together, we conclude that bTBT reduces autophagic flux via inhibition of two different steps: autophagosome formation and autophagy-lysosome fusion. A recent study showed that tributyltin (TBT), an organotin compound similar to bTBT, inhibited autophagy; however, TBT prevented lysosomal acidity, which secondary leads to autophagy inhibition, but not the autophagosome formation [30]. This indicates that the autophagy-inhibitory mechanism of TBT is different from that of bTBT. The conditions used in that study, including low TBT concentrations (300–700 nM) and different cell lines, might explain the discrepant results.

One question that remains to be answered is how does bTBT inhibit autophagosome formation? One possibility is that bTBT inhibits the function of certain autophagy proteins, including ATG2 or VMP1/TMEM41B. The autophagic structures accumulated after bTBT treatment are reminiscent as seen in ATG2 or VMP1/TMEM41B deficient cells [8,31-34]. The autophagic structures in ATG2 or VMP1/TMEM41B deficient cells were positive for LC3 and most ATG proteins including ULK1, WIPIs, DFCP1, and ATG16L. Thus, bTBT may inhibit ATG2 and VMP1/TMEM41B. Alternatively, since loss of the endosomal sorting complex required for transport -I (ESCRT-I) subunit also accumulates unclosed autophagosomal membranes with early ATG proteins [35], bTBT might inhibit the recruitment of ESCRT-I on autophagosomal membrane.

Another possibility is that bTBT inhibits the dissociation of the ATG proteins from the autophagosomes. Although the mechanism of their dissociation from the autophagosomal membrane remains unclear, early ATG proteins are released from completed autophagosome [8]. If autophagosomes containing early ATG proteins on the outer membrane fuse with lysosomes, early ATG proteins bound to the lysosomal membrane might disturb lysosomal functions due to the tightly regulated composition of lysosomal membrane proteins [36]. Actually, inhibition of LC3-PE recycling in autolysosomes was shown to induce cell death [37]. If autophagosomes containing early ATG proteins on their inner membrane fuse with lysosomes, early ATG proteins might be exhausted by degradation in lysosomes. Because autophagy is induced in nutrient starvation condition, recycling of early ATG proteins could be important to sustain autophagy under stressed conditions. Indeed, the membrane of autophagosomes contains fewer proteins compared with other organelles [38].

The third possibility is that bTBT inhibits the dephosphorylation of PI3P in phagophores during autophagosome formation. Dephosphorylation of PI3P is essential for autophagosome formation in yeast [39]. In mammals, a defect in the PI3P phosphatase MTMR3 or MTMR14 also inhibits autophagy with accumulation of LC3 and most early ATG proteins in puncta [39-41]. We speculated that bTBT might suppress PI3P phosphatase activity on the phagophore. Although a dominant negative MTMR3 mutant (inactivated mutant) has been shown to accumulate in LC3 puncta [41], MTMR3 accumulation was not detected in bTBT-treated cells in our experiment (data not shown). Detailed analysis of the bTBT mechanism of action should be further investigated.

In summary, we identified a novel autophagy inhibitor bTBT. The use of autophagy inhibitors is a promising treatment for cancer [42]. Although chloroquine and hydroxychloroquine have been used in clinical trials [20], lysosomal inhibition caused by these inhibitors has unintended side effects. If bTBT could be improved to autophagy-specific drug without affecting tubulin, then bTBT could be a useful autophagy inhibitor with a unique inhibitory mechanism for clinical application.

## Materials and Methods

### Cell culture

HeLa cells (American Type Culture Collection, ATCC), MEFs, and HEK293FT cells (Thermo Fisher Scientific) were cultured in Dulbecco’s modified Eagle’s medium (Nacalai Tesque) supplemented with 10% fetal bovine serum (MP Bio) and 50 μg/mL penicillin and streptomycin (regular medium) in a 5% CO_2_ incubator at 37°C. *ATG9A* wild-type MEFs and *ATG9A*-KO MEFs were generated previously [43]. For analysis of bTBT effects, the cells were cultured with 30 µM bTBT (Sigma-Aldrich, 251127). The cells were treated with 0.1 µM bafilomycin A_1_ (LC Laboratories, B-1080) to inhibit lysosomal degradation. The cells were incubated with 1 µM Torin 1 (Tocris Bioscience, 4247) to induce autophagy. The cells were treated with 5 µg/mL nocodazole (Cayman Chemical, 13857) and 50 µg/mL colchicine (Wako, 039-03851) to inhibit microtubule polymerization.

### Plasmids

Construction of the following plasmids was described previously: pMRX-IP GFP-ULK1, pMXs-puro GFP-DFCP1 [8], pMRXIP-GFP-Stx17 [16], and pCW RFP-GFP-LC3 [44]. To generate pLenti-puro HaloTag-HA-LC3, HaloTag-HA (synthesized by gBlocks; Integrated DNA Technologies) and LC3 (amplified from pMXs-IP-GFP-LC3) [45] were inserted into the pLenti-puro plasmid (Addgene; 17448).

### Flow cytometry

Cells detached using trypsin-EDTA were resuspended in 5% newborn calf serum containing 1 µg/mL DAPI in PBS, passed through a 70 µm cell strainer, and analyzed using the CytoFLEX S flow cytometer equipped with NUV 375-nm (DAPI), 488-nm (GFP), and 561-nm (RFP) lasers (Beckman Coulter). Dead cells were detected by DAPI staining. In each sample, 10,000 cells were acquired, and the RFP/GFP fluorescence ratio was calculated for RFP-positive cells.

### Antibodies

The rabbit polyclonal anti-LAMP1 antibody was a gift from Y. Tanaka (Kyushu University, Fukuoka, Japan). Mouse monoclonal anti-β-actin (clone 2F3, 013-24553) and mouse monoclonal anti-α-tubulin (071-25031) antibodies were purchased from Wako. Rabbit polyclonal anti-p62 (PM045), mouse monoclonal anti-LC3 (clone 8E10, M186-3) (for immunoblotting), mouse monoclonal anti-LC3 (clone 4E12, M152-3) (for immunofluorescence), and rabbit polyclonal anti-Atg16L (PM040MS) antibodies were purchased from MBL. The mouse monoclonal anti-WIPI2 (clone 2A2, MCA5780GA) antibody was purchased from Bio-Rad. Mouse monoclonal anti-HA (clone 16B12, cat. no. 901502) antibody was purchased from BioLegend. The rabbit polyclonal anti-GORASP2 (cat. no. 10598-1-AP) antibody was purchased from Proteintech.

### Immunofluorescence and fluorescence microscopy

Cells were plated on coverslips and fixed in 3.7% formaldehyde in PBS for 15 min. For immunostaining, fixed cells were permeabilized with 50 µg/mL digitonin in PBS for 5 min, blocked with 10% newborn calf serum in PBS for 10 min, and incubated with primary antibodies for 1 h. After washing three times with PBS, cells were incubated with Alexa Fluor 488-, 568-, or 647-conjugated goat anti-rabbit or anti-mouse IgG secondary antibodies (Thermo Fisher Scientific) for 30 min. The stained cells were observed under a confocal laser microscope (FV1000 IX81; Olympus) using a 60× or 100× oil-immersion objective lens with an NA of 1.40. The images were acquired using FV10-ASW 2.1 imaging software.

### Immunoblotting

For the analysis of soluble cell lysates (Figure 1D), cells were washed with cold PBS and lysed in lysis buffer (1% Triton X-100, 50 mM Tris-HCl, pH 7.5, 1 mM EDTA, 150 mM NaCl) supplemented with protease inhibitor cocktail (EDTA-free; Nacalai Tesque) and 1 mM phenylmethanesulfonyl fluoride for 15 min at 4°C. The lysates were clarified by centrifugation at 20,630 g for 5 min to remove the nuclei and were then mixed into SDS sample buffer (83 mM Tris (pH 6.8), 2% SDS, 15.5 mg/mL DTT, 5% glycerol, 0.04% bromophenol blue). For the analysis of whole cell lysates (For Figure 1E), cells were washed with cold PBS and mixed into SDS sample buffer (125 mM Tris Tris-HCL (pH 6.8), 4% SDS, 10% β-mercaptoethanol, 20% glycerol, 0.04% bromophenol blue). The samples were boiled at 95°C for 5 min. Then, 15 µg protein was added to each lane of the gel, separated by SDS/polyacrylamide gel electrophoresis, and transferred to a polyvinylidene difluoride membrane (Millipore, Billerica, MA, USA). Immunoblot analysis was performed using the indicated antibodies, and the immunoreactive bands were visualized using ImmunoStar Zeta (Wako).

### Lentiviral infection

Stable cell lines were generated using a lentiviral expression system. HEK293FT cells were transiently co-transfected with lentiviral vectors (pLenti-puro with psPAX2, Addgene, 12260 and pCMV VSV-G Addgene, 8454) using PEI MAX reagent (Polysciences, 24765-2). The medium was replaced with fresh culture medium 4 h after transfection. After culture for 72 h, the growth medium containing the lentivirus was collected and centrifuged at 2,300 g for 5 min to remove cell debris. Cells were incubated with this virus-containing medium for 48 h. Uninfected cells were eliminated using 1 µg/mL puromycin selection (InvivoGen).

### HaloTag-LC3 autophagosome completion assay

The HaloTag-LC3 autophagosome completion assay was described previously [29]. HeLa cells expressing HaloTag-LC3 were incubated in 1×MAS buffer (220 mM mannitol, 70 mM sucrose, 10 mM KH_2_PO_4_, 5 mM MgCl_2_, 2 mM HEPES, 1 mM EGTA) containing 3 nM Seahorse XF Plasma Membrane Permeabilizer (Agilent, Santa Clara, 102504-100) and 2 µM MIL (HaloTag Alexa Fluor 488 Ligand, Promega, G1001) at 37°C for 15 min. Cells were then fixed in 4% PFA for 5 min, washed three times with PBS, and incubated with 0.5 µM MPL (HaloTag TMR Ligand, Promega, G8351) for 30 min. After washing three times in PBS, the cells were observed under a confocal laser microscope.

### Statistical analysis

Quantitative data shown in the figures represent the mean ± standard deviation from three independent experiments. Statistical analyses were performed using Welch’s *t*-test or Dunnett’s test, Pearson’s chi-squared test. The Pearson’s Chi-squared test was employed to check the existence of any association with a significance level of 5%

## Supplementary Material

Supplemental Material
