# Supplementary material for: A novel autophagy inhibitor, bTBT, disturbs autophagosome formation
Source: Autophagy Rep. 2023 Apr 6;2(1):2194620. doi: 10.1080/27694127.2023.2194620 (PMC12042476; doi:10.1080/27694127.2023.2194620)
Supplement: Supplemental Material [file KAUO_A_2194620_SM4743.zip › Supplemental Figure.docx]

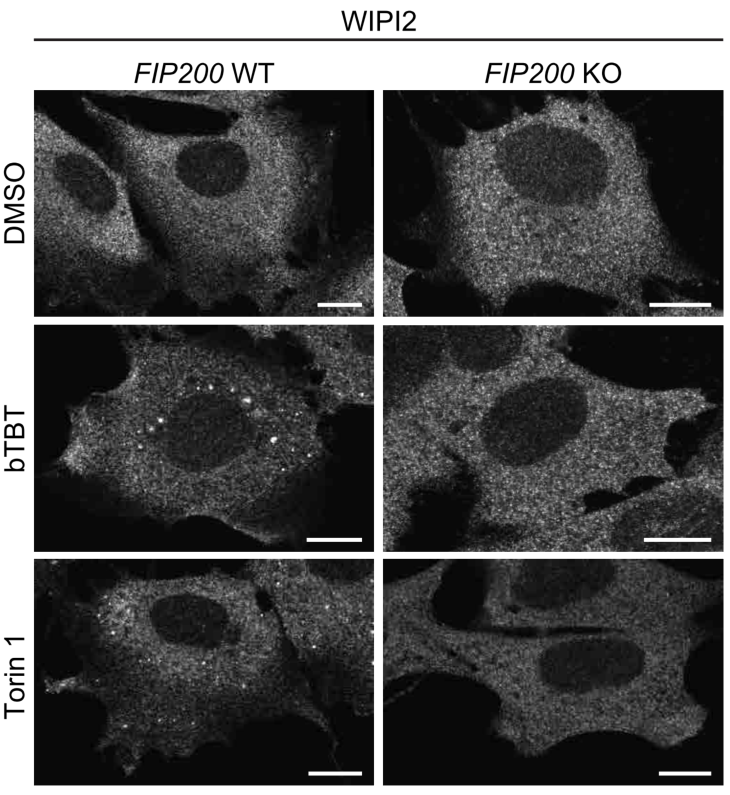


**Supplemental Figure 1. FIP200 KO MEFs does not accumulate any WIPI2 positive structures even in bTBT treatment.**

FIP200 WT or FIP200 KO MEFs were cultured with DMSO, bTBT, or Torin 1 for 2 h. Then, cells were fixed and stained with anti-WIPI2 antibodies. Immunofluorescence images were obtained using a confocal microscope. Scale bars, 10 µm.


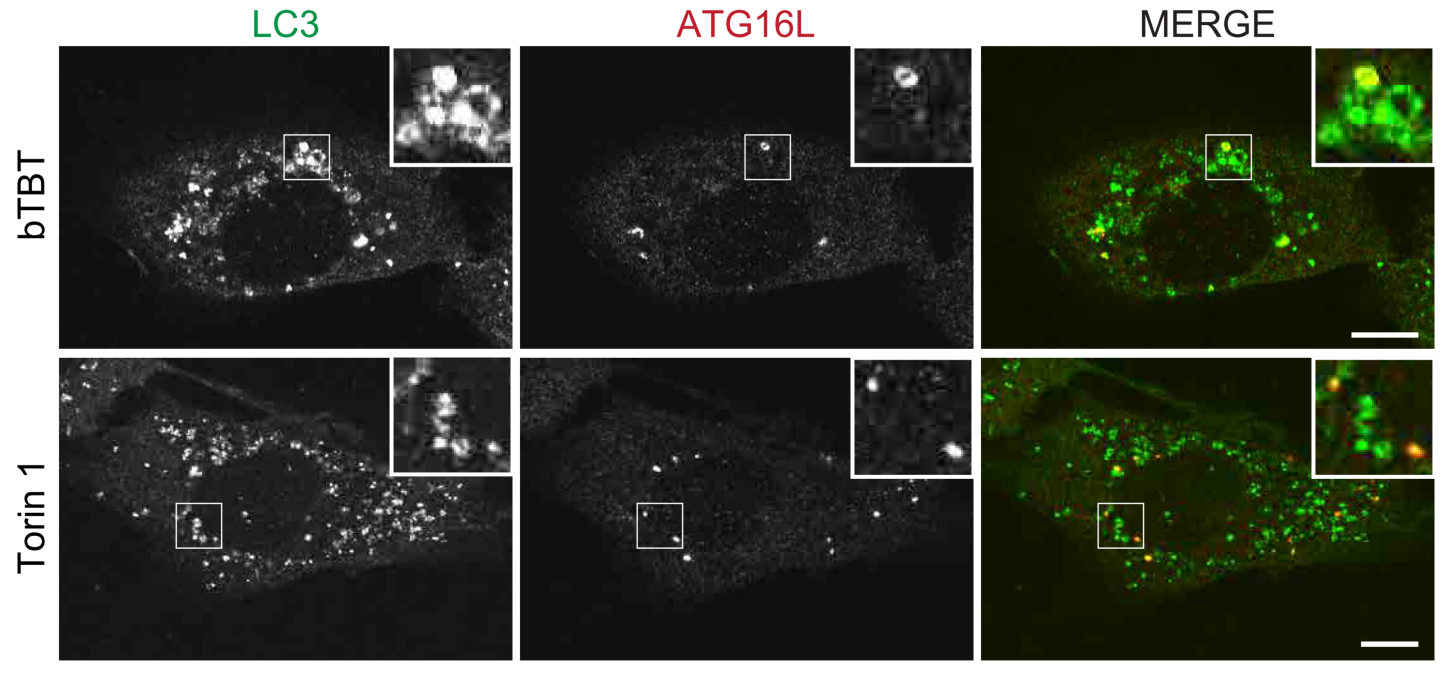

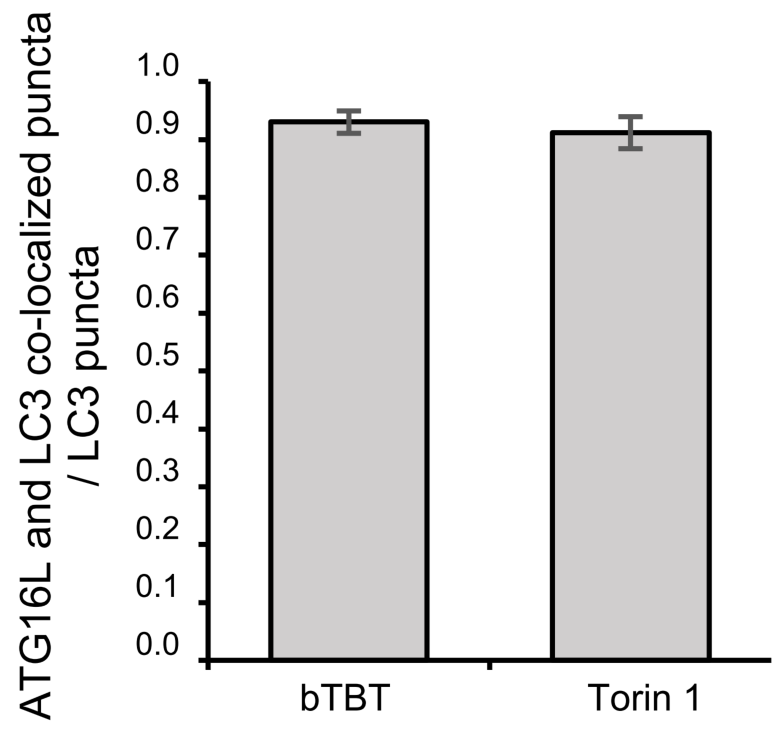


**Supplemental Figure 2. Co-localization ratio between LC3 and ATG16L.**

MEFs were cultured with bTBT or Torin 1 for 2 h. Then, cells were fixed and stained with the indicated antibodies as in Figure 2A. Co-localization ratio was analyzed by immunofluorescence images. Scale bars, 10 µm. The bar plot represents the mean ± SE of ratio in LC3 puncta with ATG16L of three independent experiments (n > 40 cells in each experiment).
